# CD4^+^ T cell count and HIV-1 viral load dynamics positively impacted by *H. pylori* infection in HIV-positive patients regardless of ART status in a high-burden setting

**DOI:** 10.1186/s40001-024-01750-6

**Published:** 2024-03-17

**Authors:** Tesfay Abadi, Takele Teklu, Tadelo Wondmagegn, Meseret Alem, Girmay Desalegn

**Affiliations:** 1 Department of Medical Laboratory Science, Adigrat University, Adigrat, Ethiopia; 2 Department of Immunology and Molecular Biology, University of Gondar, Gondar, Ethiopia; 3 Present Address: School of Medical Laboratory Sciences, College of Health Sciences and Medicine, Wolaita Sodo University, Sodo, Ethiopia; 4 Department of Medical Microbiology and Immunology, College of Health Sciences, Mekelle University, Mekelle, Ethiopia

**Keywords:** *H. pylori*, HIV, ART, CD4^+^ T cell, Viral load

## Abstract

**Background:**

There is a widespread co-infection of HIV and *Helicobacter pylori* (*H. pylori)* globally, particularly in developing countries, and it has been suggested that this co-infection may affect the course of HIV disease. However, the interplay between *H. pylori* infection and HIV disease progression is not fully elucidated. In this study, we investigated the effect of *H. pylori* co-infection on CD4^+^ T cell count and HIV viral load dynamics in HIV-positive individuals in a high co-endemic setting.

**Methods:**

A comparative cross-sectional study was conducted among 288 HIV-positive and 175 HIV-negative individuals, both with and without *H. pylori* infection. Among HIV-positive participants, 195 were on antiretroviral therapy (ART) and 93 were ART-naïve. CD4^+^ T cell count and HIV-1 viral load were measured and compared between *H. pylori*-infected and -uninfected individuals, taking into account different HIV and ART status.

**Result:**

Our study demonstrated that individuals infected with *H. pylori* had a significantly higher CD4^+^ T cell count compared to uninfected controls among both HIV-negative and HIV-positive participants, regardless of ART therapy. Conversely, HIV/*H. pylori* co-infected participants had lower HIV-1 viral load than those without *H. pylori* infection. Linear regression analysis further confirmed a positive association between *H. pylori* infection, along with other clinical factors such as BMI, ART, and duration of therapy, with CD4^+^ T cell count while indicating an inverse relationship with HIV-1 viral load in HIV-positive patients. Additionally, factors such as khat chewing, age and WHO clinical stage of HIV were associated with reduced CD4^+^ T cell count and increased HIV-1 viral load.

**Conclusion:**

Our study demonstrates that *H. pylori* co-infection was associated with higher CD4^+^ T cell count and lower HIV-1 viral load in HIV-positive patients, regardless of ART status. These findings show a positive effect of *H. pylori* co-infection on the dynamics of HIV-related immunological and virological parameters. Further studies are needed to elucidate the underlying mechanisms of the observed effects.

**Supplementary Information:**

The online version contains supplementary material available at 10.1186/s40001-024-01750-6.

## Introduction

Human immunodeficiency virus (HIV) infection remains to be one of the public health problems globally, particularly in developing countries. In 2022, approximately 39 million people globally were living with HIV, and two-thirds of these people live in sub-Saharan Africa [1]. Of these, 630,000 people died from AIDS and related illnesses in 2022. HIV is a virus that attacks the immune system, specifically targeting CD4^+^ T cells and progressively weakening the host’s ability to protect itself from opportunistic infections [2]. Following infection, the virus undergoes replication that leads to a progressive loss of CD4^+^ T cells. Thus, as the HIV infection progresses, the number of CD4^+^ T cells in the body gradually decreases [3]. Acquired immunodeficiency syndrome (AIDS) is the most advanced stage of the disease that is associated with higher HIV viral load and low CD4^+^ T cell count. Hence, when CD4^+^ T cell count starts to decline, HIV-infected individuals receive antiretroviral therapy (ART) to ensure immune recovery and prevent the depletion of more CD4^+^ T cells [4]. HIV viral load testing is crucial for monitoring HIV treatment and achieving HIV viral suppression. The World Health Organization (WHO) has identified three viral load thresholds for HIV viral suppression: unsuppressed (> 1000 copies/mL), suppressed (detected but < 1000 copies/mL), and undetectable (viral load not detected by test method) [5]. High adherence to ART is essential for achieving HIV viral suppression and ultimately reaching the goal of having an undetectable viral load.

The gastrointestinal tract (GIT) plays an important role in the clinical pathology and immunopathogenesis of HIV infection as it is the major site of HIV infection and early viral replication [6]. More than half of HIV-infected individuals present GIT symptoms and complications [7, 8]. *H. pylori* infection has been implicated to cause both protective and deleterious effects in human health and diseases [9, 10]. It has been suggested that HIV/*H. pylori* co-infections may affect the course of HIV disease progression. *H. pylori* infection is common in HIV-positive patients, especially in developing countries where both infections are prevalent, although there are conflicting findings on the prevalence of *H. pylori* infection in HIV-positive patients compared to HIV-negative individuals [11–17]. *H. pylori* is a bacterium that can colonize the lining of the gastric and duodenal mucosa and has been associated with a range of gastrointestinal diseases, including gastritis and ulcers [18]. *H. pylori* infection induces local and systemic immune responses involving a variety of immune cells, including dendritic cells (DCs), CD4^+^ Th1, CD4^+^ Th17 and regulatory T (Treg) cells [19], and these cells also play key roles in the immunopathogenesis of HIV. Nevertheless, the association between *H. pylori* infection and HIV disease progression is not fully elucidated.

In this study, we investigated the effect of *H. pylori* co-infection on the dynamics of HIV-related immunological and virological parameters in Ethiopia, where more than half of the population is infected with *H. pylori* according to a recent meta-analysis [20]. The present study demonstrated that *H. pylori* infection was significantly associated with higher CD4^+^ T cell counts and lower HIV viral loads in HIV-positive patients in a high co-endemic setting. Understanding the immunological dynamics of co-infection between HIV and *H. pylori* may provide valuable insights into improving ART efficacy and exploring alternative treatment approaches that could benefit individuals living with HIV/AIDS.

## Materials and methods

### Study design and participants

**Fig. 1:**
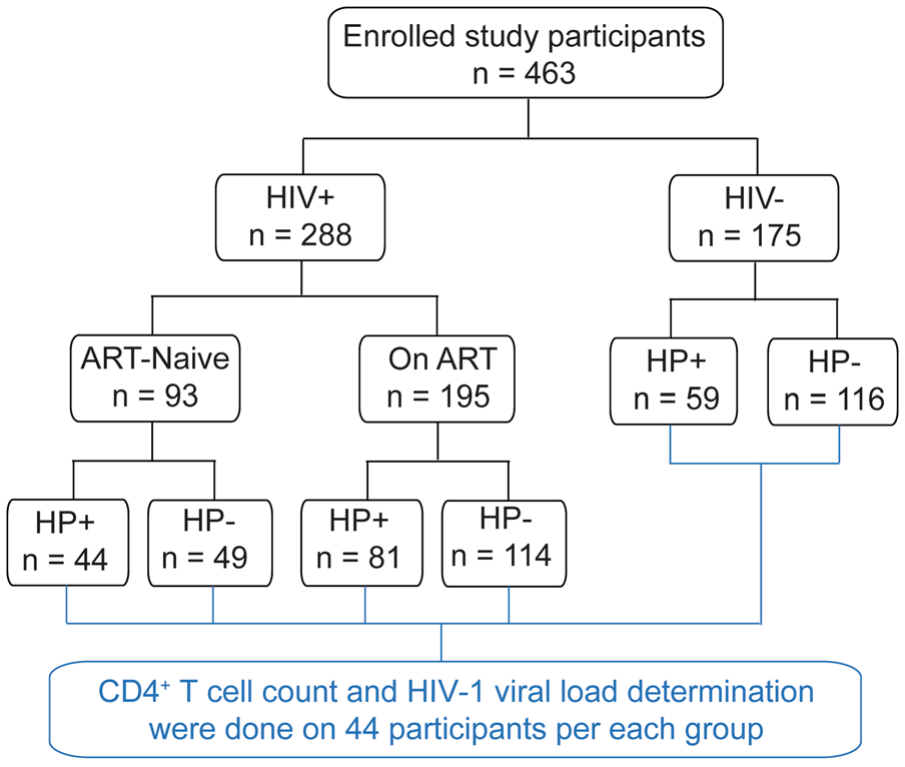
Flowchart of the enrollment of participants in this study

In this comparative cross-sectional study, study participants were recruited from randomly selected four health facilities in and around Mekelle city, Ethiopia (Mekelle General Hospital, Mekelle Health Center, Semen Health Center, and Quiha General Hospital) from June to September 2020. The recruitment and enrollment of study participants are summarized in Fig. 1. A total of 463 adult participants were consecutively recruited in this study by their attending clinicians. Volunteer participants with active tuberculosis, hepatitis, malaria, diabetes mellitus, co-infection with intestinal parasites, history of chronic disorders other than HIV and immune suppressive drugs, under 18 years old, and those who had taken anti-*H. pylori* therapy within two weeks of recruitment were excluded from the study. Enrolled study participants comprise both HIV-negative and HIV-positive individuals with and without ART therapy. Stool samples were collected from all participants for screening *H. pylori* infection. Thus, study participants were categorized into six groups based on to their clinical and treatment status (HIV, ART, and *H. pylori* status) to compare the effect of *H. pylori* co-infection on immune parameters in HIV-positive patients with and without ART. HIV-negative volunteers were also included in the study as controls.

### Data collection and laboratory examination

Socio-demographic and clinical data were collected by trained study personnel using a standardized questionnaire. Other routine clinical and laboratory parameters, including HIV status, WHO clinical stages, and ART status of the study participants, were also documented from medical records. The blood samples were collected from participants in an EDTA-containing tube for CD4^+^ T cell count and HIV viral load determination, and the stool samples were collected for screening *H. pylori* infection. HIV-1 and 2 antibody testing was also performed in certain groups to include HIV-negative participants.

#### Screening *H. pylori* infection

The fecal samples collected from the study participants were utilized to screen for *H. pylori* infection using the OneStep *H. pylori* Antigen RapiCard™ InstaTest (Cortez Diagnostics, CA). The rapid test was performed according to the kit’s instruction.

#### CD4^+^ T cell count determination

Absolute CD4^+^ T cell count (cells/µL) was analyzed using a Becton Dickinson (BD) FACSCPresto™ cell analyzer (BD, USA) at the Tigray Health Research Institute (THRI), following the established standard operating procedure (SOP).

#### HIV-1 viral RNA quantification

HIV-1 viral RNA quantification was performed using the COBAS® AmpliPrep/COBAS® TaqMan® HIV-1 Real Time PCR system (Roche Diagnostics Ltd, IN, USA) following the manufacturer’s instruction and previously described protocols [21].

### Statistical analysis

Data were entered and analyzed using SPSS 20.0 and GraphPad Prism 8.0.2. Descriptive statistics were used to describe the socio-demographic and clinical data. The normality of the data was assessed by D’Agostino and Pearson test, as well as Kolmogorov–Smirnov test. Parametric data were compared using an unpaired *t*-test and data are presented as mean ± standard deviation (SD). For non-Gaussian distributed data, non-parametric Mann–Whitney were used to compare two groups, and data are presented as median with interquartile range (IQR). Spearman’s correlation (r_s_) was used to determine the correlation between two continuous variables. Linear regression tests were used to determine the association between *H. pylori* infection and other possible risk factors, and the CD4^+^ T cell count or the HIV-1 viral load. Regression coefficient (β) and 95% confidence intervals (CI) were determined to assess the strength of associations and statistical significance. Factors with a *p* value ≤ 0.05 by the linear regression tests were included in the multiple regression analysis model. *p* ≤ 0.05 were considered statistically significant.

## Results

Out of the 463 study participants enrolled in this study, 184 (39.7%) tested positive for *H. pylori* infection while 279 (60.3%) did not have the infection (Fig. 1). Among the *H. pylori*-infected participants, 125 (67.9%) were HIV-positive and 59 (32.1%) were HIV-negative. Among the *H. pylori*-negative participants, 163 (58.4%) were HIV-positive, while 116 (41.6%) were HIV negative.

### Socio-demographic characteristics

The socio-demographic characteristics of the study participants are presented in Table 1. There were an equal proportion of male and female participants in this study, and nearly half of the *H. pylori*-infected participants being female in both HIV-positive and HIV-negative groups. The study participants ranged in age from 18 to 74 years, with a mean age of 37.2 years, and the majority fell within the 31–50 years age category in all four of group participants. Majority of the study participants were resident of urban areas (369/463, 79.7%), single (234/463, 50.5%) and had normal BMI (298/463, 64.4%) with similar proportions observed across all four groups regardless of HIV and *H. pylori* infection status. Interestingly, HIV/*H. pylori* co-infected individuals had a lower mean BMI (18.2 ± 2.4) compared to those with only *H. pylori* infection (21.9 ± 2.5). Most of the study participants had an education level of elementary school or less with virtually similar proportions among the four groups, while 53.1% (246/463) were employed and 42.1% (195/463) had low monthly income (Table 1).

**Table 1:** Socio-demographic and clinical characteristics of the study participants

Variables	No. (%) of study participants				
	HIV positive		HIV negative		Total *n* (%)
	HP + *n* (%)	HP− *n* (%)	HP + *n* (%)	HP− *n* (%)	
Gender, female	61 (48.8%)	81 (49.7%)	31 (52.5%)	53 (45.7%)	239 (51.6%)
Age (in years)					
Mean ± SD	37.2 ± 11.4	36.6 ± 11.2	36.2 ± 11.1	37.1 ± 10.9	37.2 ± 11.4
18–30	41 (32.8%)	56 (34.4%)	20 (33.9%)	35 (30.2%)	152 (32.8%)
31–50	56 (44.8%)	69 (42.3%)	27 (45.8%)	61 (52.6%)	213 (46.0%)
> 50	28 (22.4%)	38 (23.3%)	12 (20.3%)	20 (17.2%)	98 (21.2%)
Residence					
Rural	33 (26.4%)	32 (19.6%)	11 (18.6%)	18 (15.5%)	94 (20.3%)
Urban	92 (73.6%)	131 (80.4%)	48 (81.4%)	98 (84.5%)	369 (79.7%)
Marital status					
Single	60 (48.0%)	87 (53.4%)	27 (45.8%)	60 (51.7%)	234 (50.5%)
Married	42 (33.6%)	54 (33.1%)	21 (35.6%)	45 (38.8%)	162 (35.0%)
Divorced/widowed	23 (18.4%)	22 (13.5%)	11 (18.6%)	11 (9.5%)	67 (14.5%)
Educational level					
Illiterate	50 (40.0%)	54 (33.1%)	19 (32.2%)	28 (24.1%)	151 (32.6%)
Elementary School	48 (38.4%)	78 (47.9%)	30 (50.8%)	47 (40.5%)	203 (43.8%)
Secondary school and above	27 (21.6%)	31 (19.0%)	10 (16.9%)	41 (35.3%)	109 (23.5%)
Occupation					
Unemployed	66 (52.8%)	92 (56.4%)	20 (33.9%)	39 (33.6%)	217 (46.9%)
Employed	59 (47.2%)	71 (43.6%)	39 (66.1%)	77 (66.4%)	246 (53.1%)
Monthly income level					
Low	57 (45.6%)	79 (48.5%)	26 (44.1%)	33 (28.4%)	195 (42.1%)
Middle	42 (33.6%)	49 (30.1%)	25 (42.4%)	46 (39.7%)	162 (35.0%)
High	26 (20.8%)	35 (21.5%)	8 (13.6%)	37 (31.9)	106 (22.9%)
BMI (Kg/m^2^)					
Mean ± SD	18.2 ± 2.4	20.2 ± 2.5	21.9 ± 2.5	21.6 ± 2.3	20.2 ± 2.5
< 18.5	58 (46.4%)	82 (50.3%)	7 (11.9%)	18 (15.5%)	165 (35.6%)
18.5–25	67 (53.6%)	81 (49.7%)	52 (88.1%)	98 (84.5%)	298 (64.4%)

*HP* *H. pylori*, *HIV* human immunodeficiency virus, *BMI* body mass index, *SD* standard deviation

### Characteristics of clinical, immunological, and virological parameters

The clinical severity of HIV infection among the HIV-positive participants was determined based on WHO guidelines. Nearly half of them (137/288, 47.6%) were categorized as clinical stage I, with around one-third (90/288, 31.3%) classified as stage II (Table 2). The remaining participants were classified as stage III and IV. We found that most of both HIV/*H. pylori* co-infected (81/125, 64.8%) and non-co-infected HIV-positive participants (114/163, 69.9%) were on ART therapy, with the remaining being ART-naïve. The duration of ART ranged from 6 months to 20 years, with a mean (± SD) duration of 3.2 years (± 2.5), and the majority of participants (122/195, 62.6%) received therapy for less than 3 years (Table 2).

**Table 2:** Clinical, immunological, and virological parameters of the study participants

Variables	No. (%) of study participant			
	HIV positive		HIV negative *n* (%)	Total *n* (%)
	ART-naïve *n* (%)	On ART *n* (%)		
CD4^+^ T cell count (cells/µl)				
Mean ± SD	281 ± 127	442 ± 210	831 ± 106	518 ± 278
< 200	29 (33.0%)	20 (22.7%)	0 (%)	49 (18.6%)
200–500	48 (54.5%)	31 (35.2%)	0 (%)	79 (29.9%)
> 500	11 (12.5%)	37 (42.1%)	88 (100%)	136 (51.5%)
HIV-1 viral load (copies/mL)				
Median (IQR)	191,837 (15,926–350,592)	151 (75–8688)	–	11,881 (151–201,874)
< 10,000	16 (18.2%)	69 (78.4%)	–	85 (48.3%)
10,000–100,000	21 (23.8%)	17 (19.3%)	–	38 (21.6%)
> 100,000	51 (58.0%)	2 (2.3%)	–	53 (30.1%)
WHO clinical stage of HIV				
Stage I	44 (47.3%)	93 (47.7%)	–	137 (47.6%
Stage II	28 (30.1%)	62 (31.8%)	–	90 (31.3%)
Stage III	16 (17.2%)	28 (14.4%)	–	44 (15%)
Stage IV	5 (5.4%)	12 (6.2%)	–	17 (5.9%)
Duration of ART (years)				
Mean ± SD	–	3.2 ± 2.5	–	3.2 ± 2.5
< 3	–	122 (62.6%)	–	122 (62.6%)
≥ 3	–	73 (37.4%)	–	73 (37.4%)

*HIV* human immunodeficiency virus, *ART* anti-retroviral therapy, *SD* standard deviation, *IQR* interquartile range

The CD4^+^ T cell count and HIV-1 viral load, which are the most important indicators used to monitor HIV disease progression, were measured from only certain numbers of participants. The CD4^+^ T cell count measurement was done on 176 HIV-positive (88 ART-naïve and 88 on ART) and 88 HIV-negative individuals; mean ± SD: 518 ± 278. HIV-1 viral load quantification was performed only on the 176 HIV-positive participants (88 ART-naïve and 88 on ART), and the overall median (IQR) viral load was 11,881 copies/mL (151–201,874) (Table 2). As expected, HIV-positive patients had a significantly lower CD4^+^ T cell count (mean ± SD; 361 ± 191) compared to HIV-negative individuals (831 ± 106), *p* < 0.0001 (Table 2; Additional file 1: Fig. S1A). All HIV-negative participants had over 500 CD4^+^ T cells/µL, while the majority of ART-naïve (77/88, 87.5%) and ART-treated (51/88, 57.9%) HIV-positive patients had a CD4^+^ T cell count of 500/µL or lower (Table 2).

HIV-positive patients who received ART had a higher CD4^+^ T cell count (*p* < 0.0001) and lower viral load (*p* < 0.0001) compared to the ART-naïve individuals (Additional file 1: Fig. S1A, B). The inverse relationship between CD4^+^ T cell count and HIV-1 viral load in HIV-positive patients was also determined by Spearman’s correlation test (r_s_ =—0.8695, *p* < 0.0001) (Additional file 1: Fig. S2). HIV-1 viral load was then stratified into three categories: < 10,000, 10,000–100,000, and > 100,000 copies/mL (Table 2). Most of the ART-naïve participants (51/88, 58%) had a viral load above 100,000 copies/mL, while 23.8% (21/88) of them had a viral load between 10,000 and 100,000 copies/mL. In contrast, the majority of ART-treated participants (69/88, 78.4%) had a viral load below 10,000 copies/mL (Table 2). The findings align with the expected outcomes of ART in suppressing viral replication and restoring immune function.

### *H. pylori* infection was associated with higher CD4^+^ T cell count and lower HIV-1 viral load in HIV/*H. pylori* co-infected patients

**Fig. 2:**
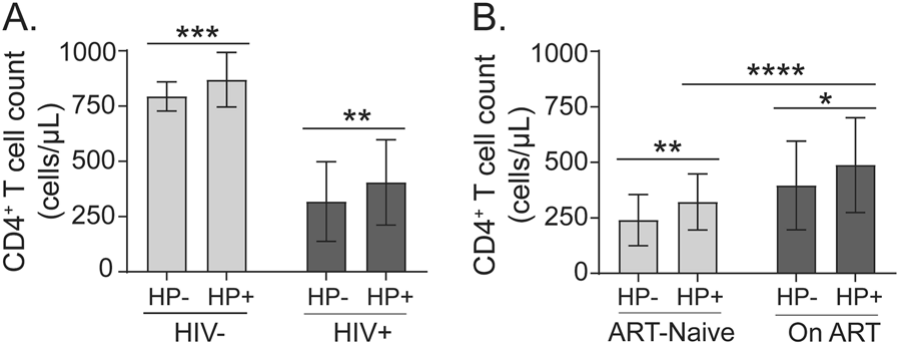
Effect of *H. pylori* infection on CD4 + T cell count. **A** Comparison of CD4^+^ T cell count between *H. pylori*-negative (HP-) and-positive (HP +) groups among HIV-negative (HIV-) and-positive (HIV +) study participants. **B** Comparison of CD4^+^ T cell count between HP- and HP + groups among ART-Naïve or on ART HIV-positive participants. *n* = 44 per group. Mean ± SD of CD4^+^ T cell counts (cells/µL) are shown. *p* values between the two groups were determined using the unpaired t-test; *p < 0.05; **p < 0.01; ***p < 0.001; ****p < 0.001

To investigate the effect of *H. pylori* infection on immunological and virological parameters dynamics, we compared CD4^+^ T cell count and HIV-1 viral load between *H. pylori* infected and uninfected participants. The present study found that *H. pylori*-infected patients had significantly higher CD4^+^ T cell count compared to those without *H. pylori* infection in both HIV-positive (mean ± SD; 405 ± 193 versus 318 ± 180; *p* = 0.0006) and HIV-negative (870 ± 124 versus 794 ± 66; *p* = 0.0024) participants (Fig. 2A). The association between *H. pylori* infection and higher CD4^+^ T cell count remained consistent across ART-naïve (*p* = 0.002) and ART-received (*p* = 0.0406) HIV-positive participants (Fig. 2B). Thus, this study provides evidence supporting the association between *H. pylori* infection and higher CD4^+^ T cell counts in HIV-positive participants, regardless of ART status.

**Fig. 3:**
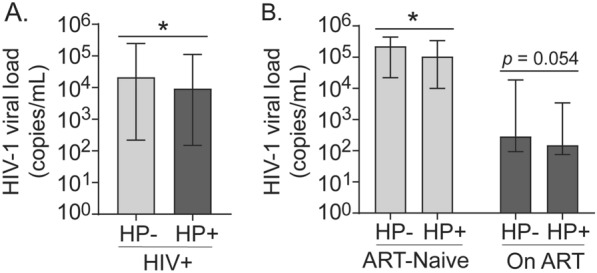
Effect of *H. pylori* infection on HIV-1 viral load level. **A** Comparison of HIV-1 viral load between *H. pylori*-negative (HP−) and-positive (HP +) groups among HIV-positive (HIV +) study participants. **B** Comparison of HIV-1 viral load between HP- and HP + groups among ART-Naïve or on ART HIV-positive participants. *n* = 44 per group. Median HIV-1 viral loads (copies/mL) with interquartile range (IQR) are shown. *p* values between the two groups were determined using the Mann–Whitney test; *p < 0.05

In addition, we assessed the association of *H. pylori* infection with HIV-1 viral load among HIV-positive participants. Interestingly, HIV-positive individuals co-infected with *H. pylori* [median (IQR); 9240 copies/mL (149—111,175)] had significantly lower viral load compared to those without *H. pylori* infection [21,271 copies/mL (219–246,895)], *p* = 0.0356 (Fig. 3A). This lower HIV-1 viral load level associated with *H. pylori* infection was also evident in both ART-naïve and ART-received groups. Specifically, in the ART-naïve participants, those infected with *H. pylori* had a substantially lower HIV-1 viral load [median (IQR); 104,919 copies/mL (9911–340,043)] compared to those who were not infected [224,472 copies/mL (21,888–442,686)], *p* = 0.0268. Among those who received ART, the median HIV-1 viral load in *H. pylori*-infected individuals [149 copies/mL (IQR: 75 – 3,409)] was lower than in those uninfected participants [287 copies/mL (IQR: 94–18,613)], although the difference was not statistically significant (*p* = 0.0536) (Fig. 3B).

Overall, this data shows that *H. pylori* co-infection was associated with higher CD4^+^ T cell count and reduced HIV-1 viral load in HIV-positive individuals, regardless of ART status. Linear regression analysis also confirmed that *H. pylori* infection was positively associated with the CD4^+^ T cell count [adjusted β (95% CI), 69.5 (11.7–127.4); *p* = 0.019], while inversely associated with the HIV-1 viral load [adjusted β (95% CI), − 20,929 (− 45,503–3,644); *p* = 0.094] in HIV-positive patients (Tables 3 and 4).

**Table 3:** Linear regression analysis of factors associated with **CD4**^**+**^** T cell count** among HIV-positive and-negative participants

Variables		No. (%) of study participants			
		HIV positive		HIV negative	
		^a^Unadjusted *β* (95% CI), *p-value*	^b^Adjusted β (95% CI), *p* value	^a^Unadjusted *β* (95% CI), *p-value*	^b^Adjusted *β* (95% CI), *p-value*
Gender, female n (%)		1.85(-55.24 to 58.94), *p* = 0.949	–	60.23(16.94 to 103.53), ***p* < 0.007**	53.79(14.41 to 93.17), ***p* = 0.008**
Age (in years)		− 3.88(− 6.48 to − 1.27), ***p* = 0.004**	− 1.60(− 4.56 to 1.35), *p* = 283	− 2.78(− 4.91 to − 0.64), ***p* = 0.011**	− 2.11(− 4.06 to − 0.15), ***p* = 0.035**
Residence					
	Rural	1		1	
	Urban	25.08(− 44.18 to 94.34), *p* = 0.476	–	23.52(− 37.97 to 85.01), *p* = 449	–
Marital status					
	Single	1		1	
	Married	− 5.32(− 68.32 to 57.68), *p* = 0.868	–	13.46(− 34.82 to 61.75), *p* = 0.581	–
	Divorced/widowed	41.86(− 44.8 to 128.52), *p* = 0.342	–	39.89(− 31.89 to 111.67), *p* = 0.272	–
Education level					
	Illiterate	1	1	1	
	Elementary school	14.75(− 48.19 to 77.68), *p* = 0.644	11.37(− 63.12 to 85.87), *p* = 0.762	39.77(− 12.16 to 91.70), *p* = 0.132	–
	Secondary school and above	105.61(15.76 to 195.46), ***p* = 0.020**	84.0(− 14.56 to 182.56), ***p* = 0.094**	43.97(− 28.27 to 116.21), *p* = 230	–
Monthly income level					
	Low	1	1	1	
	Middle	− 23.02(− 84.97 to 38.92), *p* = 0.464	28.60(− 50.71 to 107.91), *p* = 0.475	− 6.72(− 58.86 to 45.42), *p* = 0.798	–
	High	113.28(38.62 to 187.93), ***p* = 0.003**	66.34(− 17.49 to 150.18), *p* = 119	− 29.18(− 87.81 to 29.45), *p* = 0.325	
BMI (kg/m^2^)		43.72(36.05 to 51.38), ***p* < 0.001**	18.77(6.21 to 31.33), ***p* = 0.004**	11.32(1.89 to 20.75), ***p* = 0.019**	8.50(− 0.17 to 17.17), ***p* = 0.054**
*H. pylori* infection					
	Negative	1	1	1	1
	Positive	86.77(31.19 to 142.36), ***p* = 0.002**	69.54(11.70 to 127.37), ***p* = 0.019**	75.89(33.79 to 117.99), ***p* < 0.001**	64.77(25.37 to **104.16), *p* = 0.002**
Alcohol intake					
	No	1		1	
	Yes	5.25(− 59.25 to 69.76), *p* = 0.873	–	13.06(− 35.31 to 61.42), *p* = 0.593	–
Smoking habit					
	No	1		1	
	Yes	15.8(− 57.28 to 88.88), *p* = 0.670	–	− 38.28(− 97.73 to 21.17), *p* = 0.204	–
Khat chewing habit					
	No	1	1	1	
	Yes	− 132.79(− 222.59 to − 43.0), ***p* = 0.004**	− 36.00(− 128.17 to 56.16), *p* = 0.439	− 0.99(− 90.52 to 88.54), *p* = 0.983	–
WHO clinical stage of HIV					
	Stage I	1	1	N/A	
	Stage II	− 243.03(− 311.57 to − 174.5), ***p* < 0.001**	− 204.81(− 273.7 to − 135.92), ***p* < 0.001**	N/A	
	Stage III	− 360.01(− 439.5 to − 280.51), ***p* < 0.001**	− 224.96(− 332.33 to − 117.60), ***p* < 0.001**	N/A	
	Stage IV	− 453.77(− 566.97 to − 340.57), ***p* < 0.001**	− 213.01(− 379.38 to − 46.64), ***p* = 0.013**	N/A	
HIV-1 viral load (copies/mL)					
	< 10,000	1	1	N/A	
	10,000–100,000	− 247.39(− 295.9 to − 198.88), ***p* < 0.001**	− 140.06(− 241.55 to − 38.56), ***p* = 0.007**	N/A	
	> 10,0000	− 311.78(− 355.29 to − 268.27), ***p* < 0.001**	− 211.13(− 451 to 29.22), ***p* = 0.084**	N/A	
ART status					
	ART-Naïve	1	–	N/A	
	On ART	161.07(109.33 to 212.81), ***p* < 0.001**	–	N/A	
Duration of ART		1.34(0.24 to 2.44), ***p* = 0.018**	0.93(0.04 to 1.82), ***p* = 0.04**		
Duration of ART					
	< 3 years	1		N/A	
	≥ 3 years	151.94 (67.06 to 236.82), ***p* < 0.001**	82.06 (− 5.78 to 169.89), ***p* = 0.067**	N/A	

*H. pylori* *Helicobacter pylori,* *HIV* human immunodeficiency virus, *ART* antiretroviral therapy, *β* regression coefficient, *CI* confidence interval, 1 = reference category

^a^Linear regression analysis

^b^Multiple regression adjusted for factors with *p* value ≤ 0.05 by linear regression analysis

**Table 4:** Linear regression analysis of factors associated with HIV-1 viral load among HIV-positive participants

Variables	No. (%) of HIV-positive study participants	
	^a^Unadjusted β (95% CI), *p* value	^b^Adjusted β (95% CI), *p* value
Gender, female n (%)	1331,151(− 74,213 to 85,999), *p* = 0.885	–
Age (in years)	2,705 (− 1,202 to 6,433), *p* = 154	–
Residence		
Rural	1	–
Urban	− 32,293(− 129,499 to 64,914), *p* = 0.513	–
Marital status		
Single	1	1
Married	− 64,128.9 (− 151,439.3 to 231,81.6), *p* = 149	− 22,735(− 51,558 to 6,087), *p* = 0.120
Divorced/widowed	− 132,069.98 (− 252,174 to − 11,966), ***p* = 0.031**	− 10,167(− 42,010 to 21,677), *p* = 0.527
Education level		
Illiterate	1	–
Elementary school	− 28,330 (− 117,372 to 60,712), *p* = 0.531	–
Secondary school and above	107,888 (− 235,016 to 19,239), *p* = 0.096	–
Monthly income level		
Low	1	1
Middle	− 46,029 (− 134,309 to 42,250), *p* = 0.305	21,741(− 7,068 to 50,551), *p* = 0.137
High	− 145,752 (− 252,146 to − 39,359), ***p* = 0.008**	− 5,533(− 35,480 to 24,414), *p* = 714
BMI (kg/m^2^)	− 39,453 (− 52,301 to − 26,604), ***p* < 0.001**	776(− 4,093 to 5,644), *p* = 0.752
*H. pylori* infection		
Negative	1	1
Positive	− 108,027 (− 186,470 to − 29,586), ***p* = 0.007**	− 20,929(− 45,503 to 3,644), ***p* = 0.094**
Alcohol intake		
No	1	–
Yes	57,554 (− 32,550 to 147,657), *p* = 0.209	–
Smoking habit		
No	1	–
Yes	− 66,451 (− 168,565 to 35,664), *p* = 201	–
Khat chewing habit		
No	1	1
Yes	146,158 (18,982 to 273,335), ***p* = 0.025**	23.322 (− 14,820 to 61,465), *p* = 0.227
WHO clinical stage of HIV		
Stage I	1	1
Stage II	4,534 (− 20,593 to 29,661), *p* = 0.721	− 8,643(− 46,412 to 29,126), *p* = 0.650
Stage III	24,365 (− 4,782 to 53,513), *p* = 0.100	432 (− 48,453 to 49,316), *p* = 0.986
Stage IV	158,744 (117,243 to 200,246), ***p* < 0.001**	115,464(49,338 to 181,590), ***p* < 0.001**
CD4^+^ T cell count (cells/µl)		
< 200	1	1
200–500	− 301,721 (− 382,054 to − 221,388), ***p* < 0.001**	− 55,118(− 91,424 to − 18,812), ***p* = 0.003**
> 500	− 370,605 (− 460,321 to − 280,889), ***p* < 0.001**	− 49,324(− 91,609 to 7,039), ***p* = 0.023**
ART status		
ART-naïve	1	–
On ART	− 239,674 (− 311,286 to − 168,061), ***p* < 0.001**	–
Duration on ART	− 160 (− 492 to 172), *p* = 0.341	–
Duration of ART		
< 3 years	1	–
≥ 3 years	− 19,847 (− 46,207 to 6,513), *p* = 0.138	–

*H. pylori*, *Helicobacter pylori,* *HIV* human immunodeficiency virus. *ART* antiretroviral therapy, *β* regression coefficient, *CI* confidence interval; 1 = reference category

^a^Linear regression analysis

^b^Multiple regression adjusted for factors with *p* value ≤ 0.05 by linear regression analysis

### Association of socio-demographic and clinical factors with CD4^+^ T cell count and HIV-1 viral load

We further assessed the association of socio-demographic, economic and clinical factors with CD4^+^ T cell count and HIV-1 viral load parameters in HIV-positive or -negative individuals using a simple and multiple linear regression analysis and presented them in Tables 3 and 4, respectively. Socio-demographic factors, such as gender, residence area, marital status or occupation showed no significant association with CD4^+^ T cell count (Table 3) or HIV-1 viral load levels (Table 4) in HIV-positive individuals. Intriguingly, linear regression analysis demonstrated that khat chewing, a psychoactive substances, was associated with lower CD4^+^ T cell count [unadjusted β (95% CI), − 132.8 (− 222.6–− 43.0); *p* = 0.004] and higher HIV-1 viral loads [unadjusted β (95% CI), 146,158 (18,982–273,335), *p* = 0.025] in HIV-positive patients, while alcohol drinking and smoking showed no association with CD4^+^ T cell count and HIV-1 viral load regardless of HIV status. Clinical factors, such as BMI, ART status, and duration of therapy, were associated with higher CD4^+^ T cell count and lower HIV-1 viral load (Tables 3 and 4). Conversely, the WHO clinical HIV stage was negatively associated with CD4^+^ T cell count and positively related to viral load among HIV-positive individuals (Tables 3 and 4).

## Discussion

*H. pylori* infection is often considered a disease of poverty as it is associated with poor hygiene and sanitation conditions and is more prevalent in low-income and developing countries where HIV infection is rampant. We found that *H. pylori* infection rate was higher in HIV-positive patients compared to HIV-negative controls although several other studies reported a lower *H. pylori* infection prevalence in HIV-positive patients than HIV-negative individuals [11–14]. The present study investigated the effect of *H. pylori* infection on CD4^+^ T cell counts and HIV viral load levels, which are the most important and widely used predictors of progression to AIDS [22], among people infected with HIV in a high co-endemic setting. Lower CD4^+^ T cell count and higher viral loads are generally associated with more advanced stages of HIV infection and increased risk of opportunistic infections and other complications. ART, which is the standard treatment for HIV infection, suppresses viral replication, exert immune restoration, and stall the progression of HIV to AIDS [4, 23, 24], demonstrating the positive health outcomes of ART among people living with HIV. Interestingly, this study demonstrates that *H. pylori* co-infection was positively associated with elevated CD4^+^ T cell counts and reduced HIV viral load in HIV-positive patients, consistent with a previous study [25]. The favorable immunological and virological parameters in relation to *H. pylori* positivity persisted among individuals who initiated ART.

The inverse correlation between *H. pylori* infection and immunosuppression among HIV-positive patients has become a subject of scientific interest. Several theories and mechanisms have been proposed to explain this association, although the precise mechanisms are not fully understood. Firstly, *H. pylori*, as its immune evasion strategy, interferes with T cell activation to maintain its persistence and pathogenesis in the gastric and intestinal mucosa [26–29]. Persistent activation of CD4^+^ T cells contributes to progressive loss of CD4^+^ T cells during HIV infection. Increased immune activation markers have also been observed in HIV-positive patients receiving ART, despite suppression of viral replication and CD4^+^ T cell reconstitution [30]. Thus, the diminished CD4^+^ T cell activation during *H. pylori* infection may affect susceptibility of target cells for HIV infection, slowing replication and distribution of the virus. This suggest that *H. pylori* infection plays a protective role against HIV infection through a mechanism different from that of ART.

The immune response to *H. pylori* is predominantly CD4^+^ Th1 or CD4^+^ Th17-mediated inflammatory responses [19, 31]. However, this host immune response is unable to clear the bacteria, resulting in a lifelong persistence. The underlying regulatory mechanism of its lifelong *H. pylori* colonization in the human stomach could involve production of Treg cells. *H. pylori* actively targets DCs and interferes its maturation and antigen presentation in a way that promotes the generation of Treg cells in the gut-associated lymphoid tissue (GALT) [19, 32, 33]. These Treg cells secrete cytokines that inhibits CD4^+^ Th1 or CD4^+^ Th17 effector functions [33]. In individuals infected with *H. pylori*, gastric lymphocytes exhibit elevated production of IL-10 while displaying reduced levels of IFN-γ compared to those not infected with *H. pylori* [34, 35]. Moreover, several studies reported the protective immunomodulatory properties of *H. pylori* against allergic asthma [36–38] and chronic inflammatory disorder [9, 38]. *H. pylori* infection inhibits these chronic disorders by targeting DCs and regulating the ratio between the pro-inflammatory CD4^+^ Th1 and CD4^+^ Th17 cells, and the tolerogenic Treg cells [38–40]. This indicates that *H. pylori* might employ immune evasion mechanism that could be beneficial in fighting against other chronic infection and disorders.

Several socio-demographic and clinical factors were related to immunological and virological parameters in HIV-positive patients. Clinical factors such as BMI, ART status, and duration of therapy were positively associated with CD4^+^ T cell count while inversely related to HIV-1 viral load in HIV-positive patients. The use of ART is associated with CD4^+^ T cells recovery in HIV-positive patients and the change rate increased with duration of therapy [41]. Age and WHO clinical stage of HIV were negatively associated with reduced CD4^+^ T cell count and increased HIV-1 viral load, consistent with other studies [42]. Interestingly, the habit of khat chewing had a deleterious effect on the CD4^+^ T cell count and HIV-1 viral load dynamics of HIV-positive individuals.

## Conclusion

This study demonstrated that *H. pylori* infection correlates with higher CD4^+^ T count and lower HIV-1 viral load, demonstrating favorable immunological and virological parameters in HIV-positive individuals regardless of ART therapy. These findings contribute to our understanding of the intricate interplay between HIV, *H. pylori* and the immune system. Nevertheless, further studies are needed to investigate the precise mechanisms underlying these interactions and the prospect of potential therapeutic approaches.

### Supplementary Information

**Additional file 1: Fig. S1.** CD4^+^ T cell count and HIV viral load in study participants.** A** Comparison of CD4^+^ T cell count between HIV-negative (HIV-) and-positive (HIV+) study participants, and between ART-naïve and ART-received HIV-positive participants. n = 44 per group. Mean + SD of CD4^+^ T cell counts (cells/µL) are shown. **B** Comparison of HIV-1 viral load between ART-Naïve or ART-received HIV-positive participants. *n*=44 per group. Median HIV-1 viral loads (copies/mL) with interquartile range (IQR) are shown. *p* values between the two groups were determined using the unpaired t-test; ****p<0.001. **Fig. S2. **Correlation between CD4^+^ T cell count and HIV viral load in HIV-positive participants was determined using Spearman correlation (*r*_*s*_); *n* = 174. *p* values are shown.

## Data Availability

The datasets used and analyzed during the current study are available from the corresponding author on reasonable request.
