# Supplementary material for: CD4+ T cell count and HIV-1 viral load dynamics positively impacted by H. pylori infection in HIV-positive patients regardless of ART status in a high-burden setting
Source: Eur J Med Res. 2024 Mar 17;29:178. doi: 10.1186/s40001-024-01750-6 (PMC10946129; doi:10.1186/s40001-024-01750-6)
Supplement: Supplementary file 1 — Additional file 1: Fig. S1. CD4+ T cell count and HIV viral load in study participants. A Comparison of CD4+ T cell count between HIV-negative (HIV-) and-positive (HIV+) study participants, and between ART-naïve and ART-received HIV-positive participants. n = 44 per group. Mean + SD of CD4+ T cell counts (cells/µL) are shown. B Comparison of HIV-1 viral load between ART-Naïve or ART-received HIV-positive participants. n=44 per group. Median HIV-1 viral loads (copies/mL) with interquartile range (IQR) are shown. p values between the two groups were determined using the unpaired t-test; ****p<0.001. Fig. S2. Correlation between CD4+ T cell count and HIV viral load in HIV-positive participants was determined using Spearman correlation (rs); n = 174. p values are shown. [file 40001_2024_1750_MOESM1_ESM.docx]

**Additional file 1**


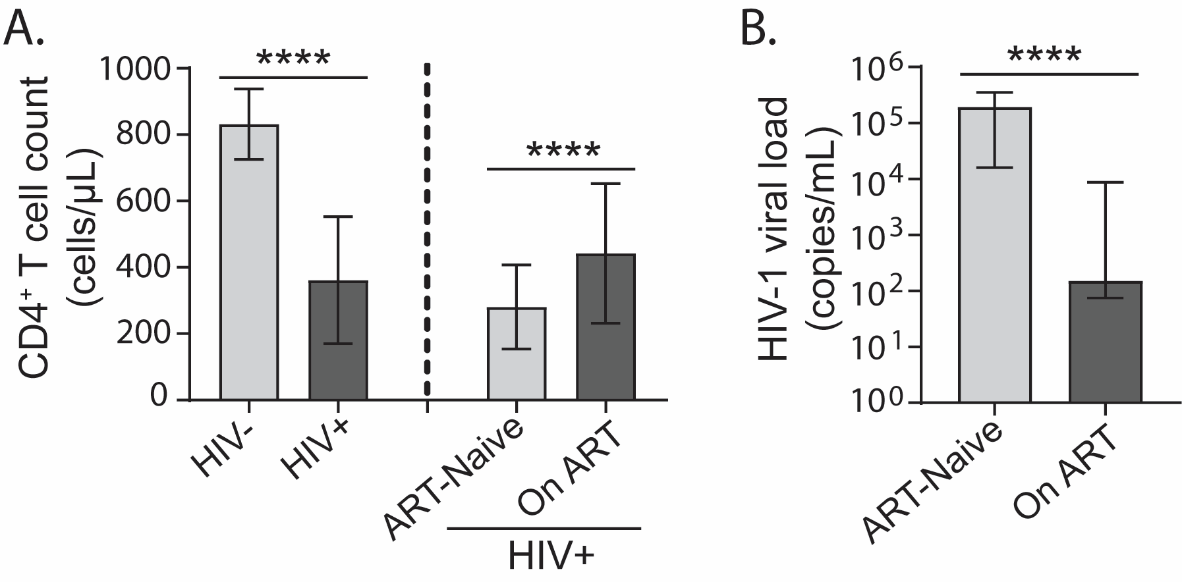


**Fig. S1 CD4^+^ T cell count and HIV viral load in study participants.** A) Comparison of CD4^+^ T cell count between HIV-negative (HIV-) and-positive (HIV+) study participants, and between ART-naïve and ART-received HIV-positive participants. n = 44 per group. Mean + SD of CD4^+^ T cell counts (cells/µL) are shown. B) Comparison of HIV-1 viral load between ART-Naïve or ART-received HIV-positive participants. n = 44 per group. Median HIV-1 viral loads (copies/mL) with interquartile range (IQR) are shown. *p* values between the two groups were determined using the unpaired t-test; **** *p* < 0.001.


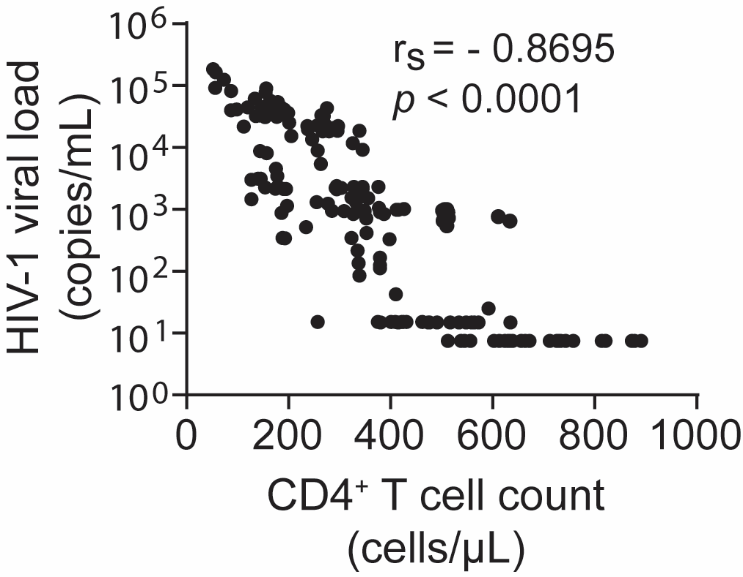


**Fig. S2** **Correlation between CD4^+^ T cell count and HIV viral load** in HIV-positive participants was determined using Spearman correlation (r_s_); n = 174. *p* values are shown.
